# Serum *Wisteria Floribunda* Agglutinin-Positive Sialylated Mucin 1 as a Marker of Progenitor/Biliary Features in Hepatocellular Carcinoma

**DOI:** 10.1038/s41598-017-00357-8

**Published:** 2017-03-21

**Authors:** Nobuharu Tamaki, Atsushi Kuno, Atsushi Matsuda, Hanako Tsujikawa, Ken Yamazaki, Yutaka Yasui, Kaoru Tsuchiya, Hiroyuki Nakanishi, Jun Itakura, Masaaki Korenaga, Masashi Mizokami, Masayuki Kurosaki, Michiie Sakamoto, Hisashi Narimatsu, Namiki Izumi

**Affiliations:** 1 Department of Gastroenterology and Hepatology, Musashino Red Cross Hospital, Tokyo, Japan; 2 Research Center for Medical Glycoscience, National Institute of Advanced Industrial Science and Technology, Ibaraki, Japan; 3 Department of Pathology, Keio University School of medicine, Tokyo, Japan; 4 Research Center for Hepatitis and Immunology, National Center for Global Health and Medicine, Chiba, Japan

## Abstract

Histological molecular classification of hepatocellular carcinoma (HCC) is clinically important for predicting the prognosis. However, a reliable serum marker has not been established. The aim of this study was to evaluate the diagnostic value of serum *Wisteria Floribunda* agglutinin-positive sialylated mucin 1 (WFA-sialylated MUC1), which is a novel biliary marker, as a marker of HCC with hepatic progenitor cell (HPC)/biliary features and of prognosis. A total of 144 consecutive patients who underwent complete radiofrequency ablation of primary HCC were enrolled. A serum WFA-sialylated MUC1 level of 900 μL/mL was determined as the optimal cutoff value for prediction of immunohistochemical staining for HPC/biliary features [sialylated MUC1 and cytokeratin 19 (CK19)]. Positive staining rate of sialylated MUC1 and CK19 was significantly higher in patients with WFA-sialylated MUC1 ≥900 than those with WFA-sialylated MUC1 <900. Furthermore, cumulative incidence of HCC recurrence was significantly higher in patients with WFA-sialylated MUC1 ≥900 and on multivariate analysis, serum WFA-sialylated MUC1 levels was an independent predictor of HCC recurrence. These results revealed that serum WFA-sialylated MUC1 was associated with histological feature of HCC and recurrence after curative therapy and it could be a novel marker of HPC/biliary features in HCC and of prognosis.

## Introduction

Hepatocellular carcinoma (HCC) is one of the most common malignant neoplasms in the world^1^. Because of recent progress in curative therapy with surgical resection or radiofrequency ablation (RFA), 5-year survival rates are over 60%–70% for early stage disease^2^. However, approximately 70% of patients will have recurrence within 5 years of curative therapy. Therefore, prediction of HCC recurrence is an important issue^3^.

Recently, molecular classification of HCC had been advocated because it has been correlated with clinical outcome and may have clinical value as a predictive biomarker to guide therapeutic decision^4, 5^. Several studies reported that some HCCs originate from hepatic progenitor cells (HPC)^6, 7^. The cells in such tumors are thought to express both hepatic and biliary features and feature heterogeneous differentiation^8^. Those subtypes of HCCs with HPC/biliary features have been associated with more aggressive biological characteristics, including recurrence and metastasis. Cytokeratin 19 (CK19) is known as a marker of HPC/biliary features and the expression of CK19 in HCC tissue has been linked to a poor prognosis^9–13^. Furthermore, mucin-1 (MUC1) is also known as a biliary marker in HCC tissues and the expression of MUC1 in HCC is also associated with a poor prognosis^14, 15^. Therefore, histological molecular classification of HCC tumors is clinically relevant for predicting the prognosis. However, this requires either surgical resection or tumor biopsy for pathological diagnosis and, to date, a reliable serum marker to reflect HPC/biliary features of HCC and replace pathological diagnosis has not been established.

*Wisteria Floribunda* agglutinin-positive sialylated mucin 1 (WFA-sialylated MUC1) is a new, sensitive biliary marker for human cholangiocarcinoma. The diagnostic utility of WFA-sialylated MUC1 for cholangiocarcinoma has been reported, either with histochemical staining or detection in bile^16, 17^. In addition, a method to measure WFA-sialylated MUC1 in serum samples has recently been established, yielding high diagnostic performance for cholangiocarcinoma^18, 19^. Histochemical WFA-sialylated MUC1-positive staining is observed in a proportion of HCCs with biliary features^16^. However, the association between serum WFA-sialylated MUC1 and histochemical features in HCC is unclear. Here, we hypothesized that serum WFA-sialylated MUC1 has diagnostic value to reflect the expression of biliary feature in HCC nodules and it might, therefore, be a useful predictive marker of subtypes of HCCs with HPC/biliary features, possibly obviating the need for histochemical diagnosis. High serum levels of WFA-sialylated MUC1 might then also suggest a poor prognosis, even after curative therapy. The aim of this study was to evaluate whether serum WFA-sialylated MUC1 levels reflect positive staining of CK19 and sialylated MUC-1 in HCCs and to determine the association of serum WFA-sialylated MUC1 levels and the clinical course after curative therapy.

## Results

### Patient characteristics and immunohistochemical studies

**Figure 1:**
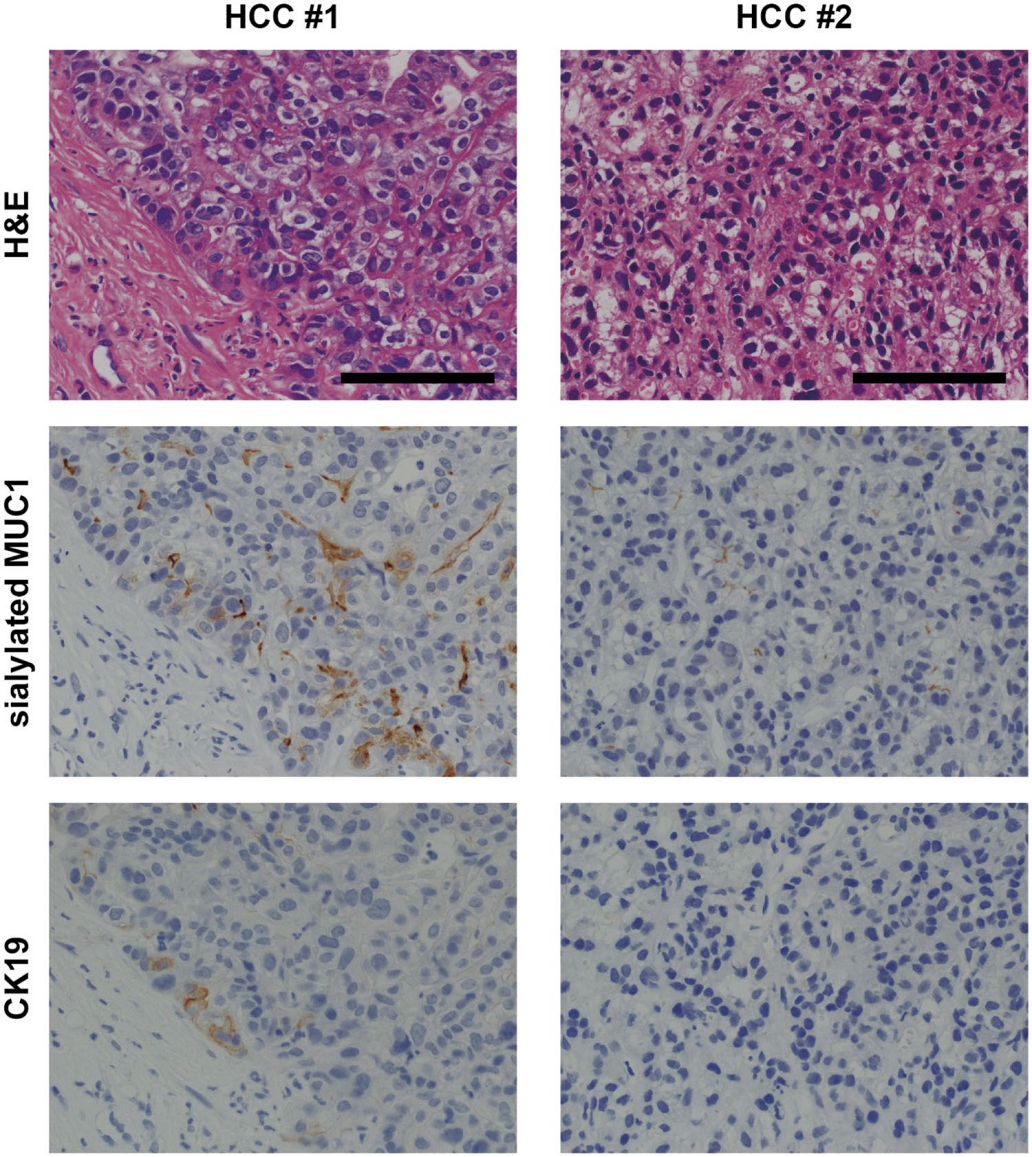
Representative staining for sialylated MUC1 and CK19 in liver biopsies. Liver biopsies (HCCs #1 and #2) were stained with hematoxylin–eosin (H&E) and antibodies against sialylated MUC1 and CK19. It was regarded as sialylated MUC1-positive in HCCs #1 and #2, while CK19-positive in HCC #1 and –negative in HCC #2. Scale bar = 100 µm.

**Figure 2:**
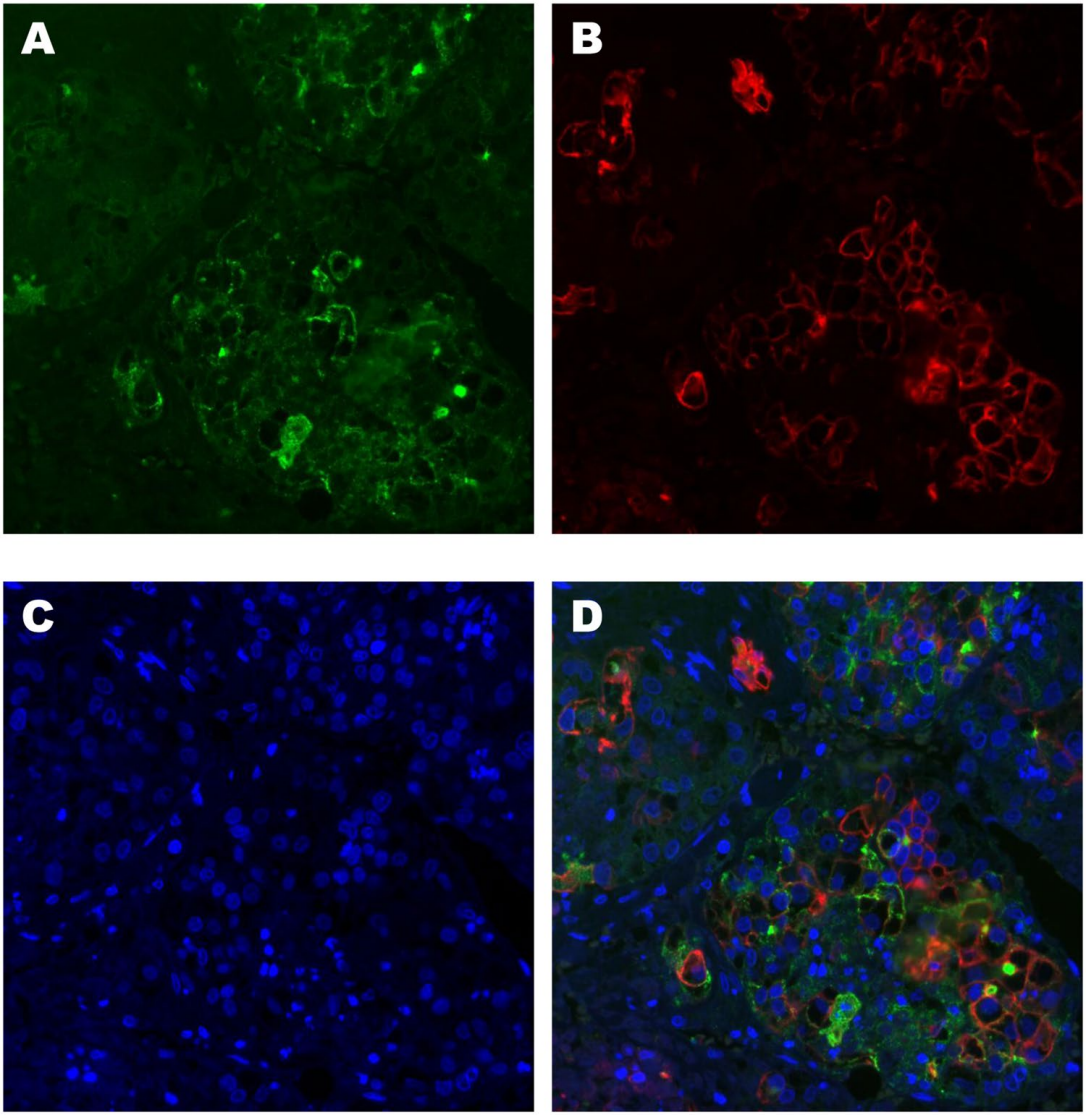
Double-staining for sialylated MUC1 and CK19. In sialylated MUC1 (green) positive tumor cells (**A**), CK19 (red) was also detected (**B**). The nucleus (blue) was stained with Hoechst 33342 (**C**). The merged image (**D**) showed coexpression of sialylated MUC1 and CK19 in some tumor cells.

Patient characteristics are shown in Table 1. All patients were treated with RFA and all had imaging confirmation of complete ablation. Tumor biopsy samples were obtained before RFA. The mean WFA-sialylated MUC1 level was 334 μL/mL (range, 27 to 3190 μL/mL). Of the 61 tumor biopsy specimens, sialylated MUC1- and CK19-positive immunohistochemical staining was positive in 16% (10/61) and 10% (6/61), respectively (Fig. 1). Sialylated MUC1 was predominantly localized on the bile canalicular surface of tumor cells. In non-tumor hepatocytes and stromal cells, sialylated MUC1 was not detected. All of the CK19 positive samples were also positive for sialylated MUC1. In CK19 and sialylated MUC1 positive sample, fluorescence double-staining indicated that coexpression of CK19 and sialylated MUC1 was confirmed in some tumor cells (Fig. 2).

**Table 1:** Patient’s characteristics (n = 144).

Age, years	72.2 ± 10.0
Sex, male/female	86/58
Etiology, HCV/HBV/Others	105/11/28
AST, IU/ml	50.6 ± 24.1
ALT, IU/ml	43.2 ± 26.2
Albumin, g/dl	3.6 ± 0.5
Bilirubin, mg/dl	0.9 ± 0.4
AFP, ng/ml	11.7 (2–3290)
AFP-L3, %	4.1 (0.5–79.9)
DCP, mAU/ml	26.5 (10–11200)
WFA- sialylated MUC1, μL/mL	334 (27–3190)
Child-Pugh, A/B	131/13
Tumor size, <20 mm/≥20 mm	70/74
Tumor number, single/2–3	109/35
Histological findings (n = 61)	
Differentiation, well/moderate/poor	16/41/4
Sialylated MUC-1, positive/negative	10/51
CK19, positive/negative	6/55

### Association between serum WFA-sialylated MUC1 and immunohistochemical staining

**Figure 3:**
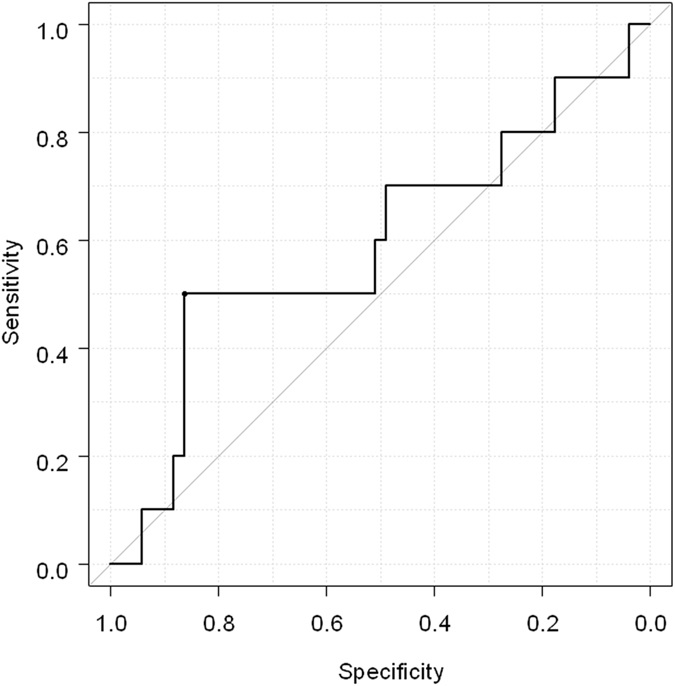
ROC analysis of serum WFA-sialylated MUC1 for detection of sialylated MUC1 positive staining.

Serum levels of WFA-sialylated MUC1 were analyzed based on the results of immunohistochemical staining. For patients with sialylated MUC1-positive staining, based on the ROC analysis, AUROC of WFA-sialylated MUC1 level was 0.60 and a WFA-sialylated MUC1 level of 900 μL/mL was selected as the optimal cutoff value (Fig. 3). For those with CK19-positive staining, the same cutoff value was selected by the ROC analysis. Sialylated MUC1-positive staining was observed in 42% (5/12) and 10% (5/49) of patients with WFA-sialylated MUC1 ≥900 μL/mL and WFA-sialylated MUC1 <900 μL/mL, respectively. Staining positivity was significantly higher in patients with WFA-sialylated MUC1 ≥900 μL/mL than in those with WFA-sialylated MUC1 <900 μL/mL (p = 0.008, Table 2). Similarly, rates of CK19 positive staining were significantly higher in patients with WFA-sialylated MUC1 ≥900 μL/mL than those with WFA-sialylated MUC1 <900 μL/mL [25% (3/12) and 6% (3/49), p = 0.04]. The positive predictive value (PPV) and negative predictive value (NPV) of WFA-sialylated MUC1 for prediction of sialylated MUC1-positive staining were 42% and 90%. Similarly, the PPV and NPV of WFA-sialylated MUC1 for prediction of CK19-positive staining were 25% and 94%. There was a significant association between serum WFA-sialylated MUC1 levels and immunohistochemical sialylated MUC-1 and CK19 positivity.

**Table 2:** Association between serum WFA-sialylated MUC1 and immunohistochemical staining.

	Sialylated MUC1 positive staining	Sialylated MUC1 negative staining	Total	
WFA-sialylated MUC1 ≥900 μL/mL	5	7	12	PPV:42%
WFA-sialylated MUC1 <900 μL/mL	5	44	49	NPV:90%
Total	10	51	61	
	Sensitivity:50%	Specificity:86%		p value = 0.008
	**CK19 positive staining**	**CK19 negative staining**	**Total**	
WFA-sialylated MUC1 ≥900 μL/mL	3	9	12	PPV:25%
WFA-sialylated MUC1 <900 μL/mL	3	46	49	NPV:94%
Total	6	55	61	
	Sensitivity:50%	Specificity:84%		p value = 0.04

### Association between serum WFA-sialylated MUC1 and other serum tumor markers

**Figure 4:**
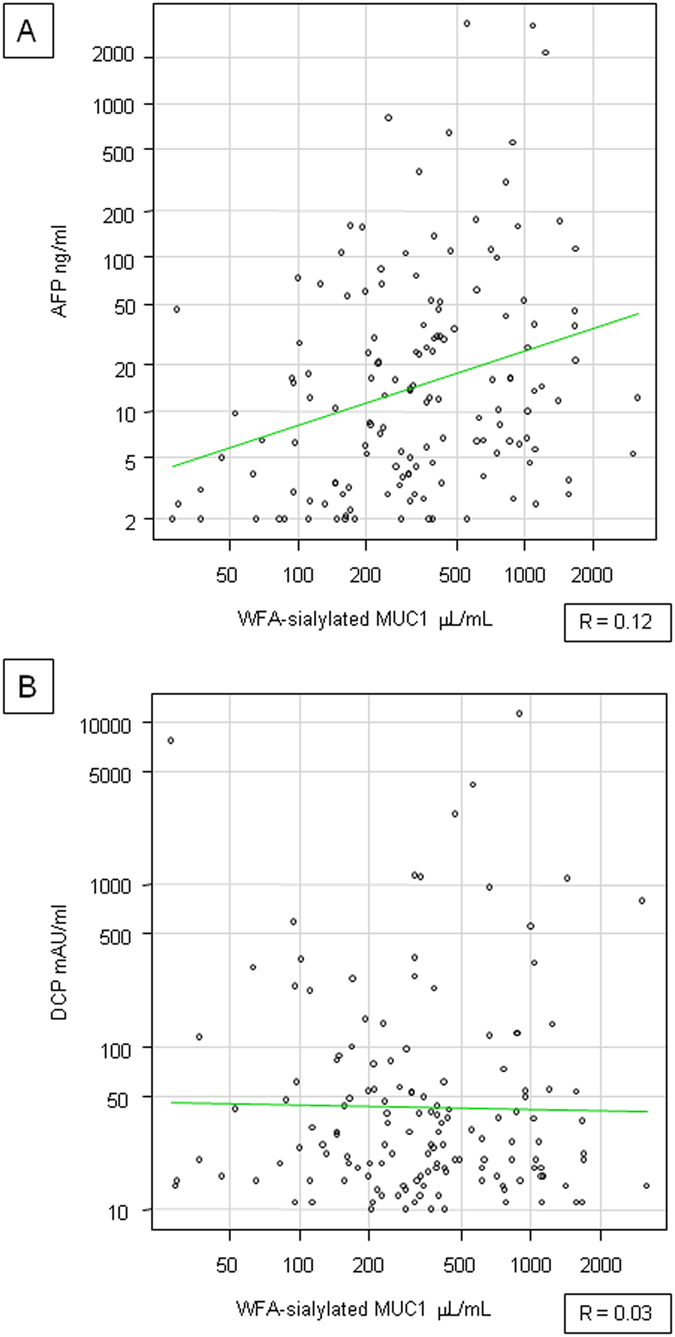
Correlation between serum WFA-sialylated MUC1 and other serum tumor markers. (**A**) Serum WFA-sialylated MUC1 and AFP (**B**) Serum WFA-sialylated MUC1 and DCP.

Comparing WFA-sialylated MUC1 with AFP, the correlation coefficient was 0.12, indicating no significant relationship (p = 0.15, Fig. 4A). Similarly, WFA-sialylated MUC1 and DCP were not significantly correlated (correlation coefficient 0.03, p = 0.73, Fig. 4B). Therefore, WFA-sialylated MUC1 levels were independent of AFP or DCP.

### HCC recurrence after curative therapy

**Figure 5:**
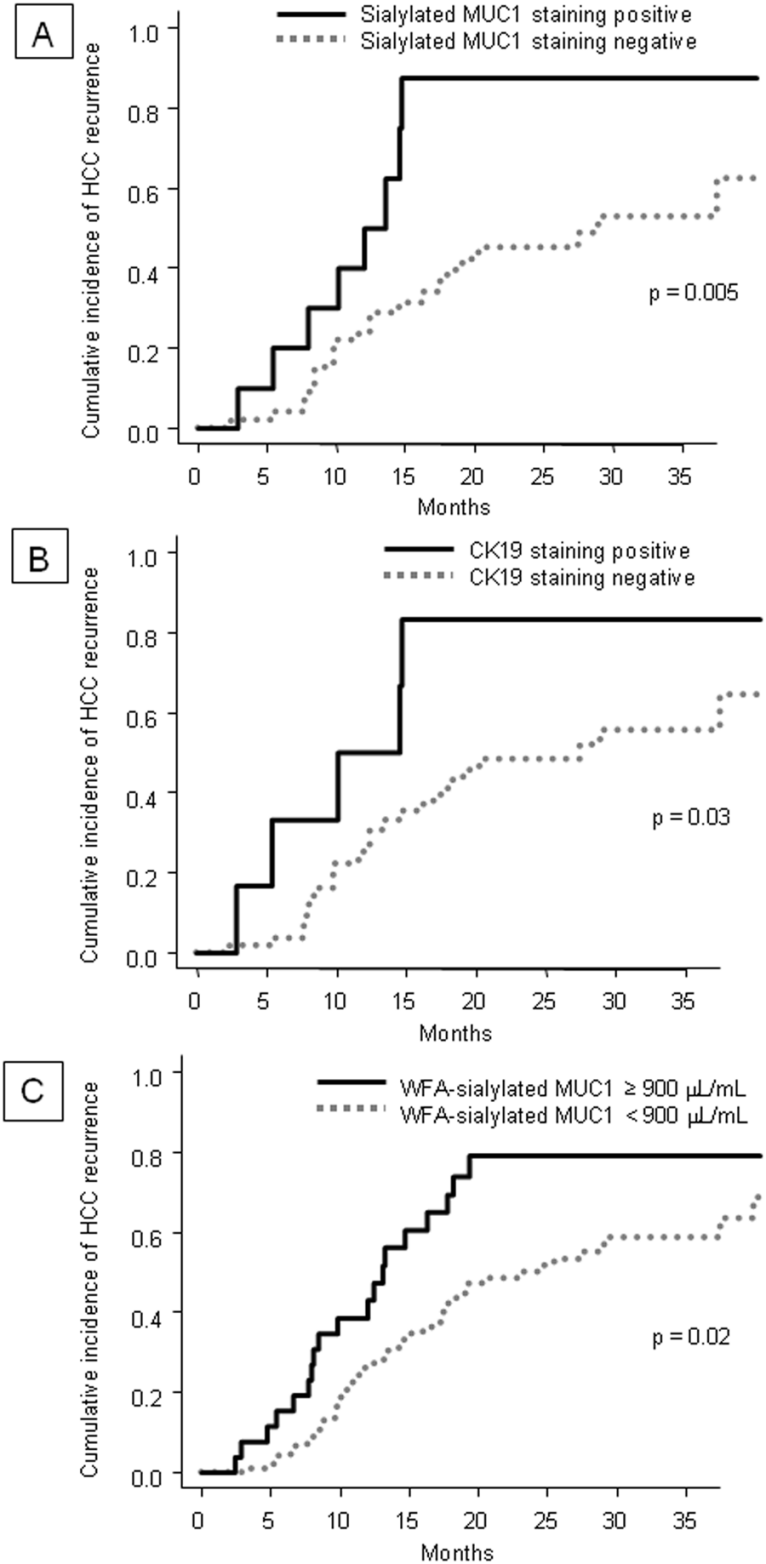
Cumulative incidence of HCC recurrence. Patients were categorized into two groups according to (**A**) sialylated MUC1 staining, (**B**) CK19 staining and (**C**) serum WFA-sialylated MUC1.

The cumulative incidence of HCC recurrence was compared with the results of initial immunohistochemical staining for sialylated MUC1 and CK19 and with serum WFA-sialylated MUC1 levels. The 1- and 3-year cumulative recurrence incidences were 50.0% and 87.5%, respectively, in patients with sialylated MUC1-positive tumors and were significantly higher than in those with sialylated MUC1-negative tumors (24.1 and 52.7%, p = 0.005, Fig. 5A). For patients with CK19-positive staining, the 1- and 3-year cumulative recurrence rates were 50.0% and 83.3%, whereas they were 26.3% and 55.4% in those with CK19-negative tumors (p = 0.03, Fig. 5B). The 1- and 3-year cumulative recurrence rates in patients with initial serum WFA-sialylated MUC1 levels ≥900 μL/mL were 42.9% and 78.9%, significantly higher than in those with WFA-sialylated MUC1 levels <900 μL/mL (26.1 and 58.6%, p = 0.02, Fig. 5C). In patients with WFA-sialylated MUC1 levels <900 μL/mL, 83% of patients had recurrence of HCC at BCLC stage 0 or A. On the other hand, in patients with WFA-sialylated MUC1 levels ≥900 μL/mL, 42% of patients had recurrence of HCC at BLCL stage B, C, or D, and these patients had poor recurrence of HCC (p = 0.02).

### Factors associated with HCC recurrence

Univariate and multivariate analysis revealed the factors that increased the hazard ratio (HR) for HCC recurrence (Table 3). Serum WFA-sialylated MUC1 was associated with HCC recurrence on univariate analysis, along with tumor size and DCP levels. On multivariate analysis, WFA-sialylated MUC1 (HR = 1.95, 95% CI: 1.15–3.29, p = 0.01) and tumor size (HR = 1.72, 95% CI: 1.04–2.82, p = 0.03) were independent predictors of HCC recurrence.

**Table 3:** Factors associated with HCC recurrence.

		Univariate			Multivariate		
		Hazard ratio	95% CI	p Value	Hazard ratio	95% CI	p Value
Age (10 year divisions)		1.09	0.87–1.35	0.4			
Sex	male	1					
	female	0.83	0.52–1.31	0.8			
Etiology	HCV	1					
	HBV/Ohters	1.11	0.67–1.81	0.7			
AST, IU/ml	<40	1					
	≥40	0.99	0.63–1.55	0.9			
ALT, IU/ml	<40	1					
	≥40	0.84	0.53–1.32	0.5			
Albumin, mg/dl		0.75	0.52–1.07	0.1			
Bilirubin, mg/dl		0.88	0.51–1.51	0.6			
AFP, ng/ml	<10	1					
	≥10	1.29	0.82–2.03	0.3			
AFP-L3, %	<10	1					
	≥10	1.58	0.88–2.84	0.1			
DCP, mAU/ml	<40	1			1		
	≥40	1.89	1.20–2.97	0.006	1.57	0.95–2.58	0.07
WFA-sialylated MUC1, μL/mL	<900	1			1		
	≥900	1.82	1.08–3.08	0.02	1.95	1.15–3.29	0.01
Child-Pugh	B	1					
	A	0.92	0.37–2.30	0.9			
Tumor size, mm	<20	1			1		
	≥20	2.00	1.27–3.16	0.002	1.72	1.04–2.82	0.03
Tumor number	single	1					
	multiple	1.18	0.72–1.94	0.5			

## Discussion

In this study, we found that serum WFA-sialylated MUC1 levels constitute a reliable serum marker of a subtype of HCC with HPC/biliary features. These features were not only associated with positive histochemical staining for CK19 and sialylated MUC1 but also with an increased risk of HCC recurrence after RFA therapy with curative intent. These findings indicate that serum WFA-sialylated MUC1 could be used as a non-invasive biomarker of aggressiveness of HCC. Immunohistochemical staining for CK19 and MUC1 are known as markers of HPC/biliary features and are associated with a poor prognosis after curative therapy^9–15^. However, a reliable serum marker to reflect HPC/biliary features of HCC remains to be established. Serum WFA-sialylated MUC1 was evaluated as a marker of a subtype of HCC with HPC/biliary features and a risk of HCC recurrence. Because serum WFA-sialylated MUC1 measurement is non-invasive and easily performed, it may have a great impact on classification of HCC in clinical practice.

MUC1 plays a key role as an oncogene in tumorigenesis and some studies have shown that MUC1 is overexpressed in human HCC tissue^20–23^. Although MUC1 itself is widely used as a histochemical or serological diagnostic marker of various cancers^24^, the diagnostic value and specificity of MUC1 tend to be limited because the level of MUC1 expression is similar in normal and tumor cells. MUC1 is known as a highly glycosylated mucin associated with malignancy in many organs^25^. Its glycosylation pattern is altered with the progression of disease and aberrant glycosylation is often associated with individual steps in disease progression^26, 27^. Therefore, the detection of disease-associated modification of glycosylation patterns is an important step in the diagnosis of cancers and glycoproteins that exhibit disease-associated modification of glycosylation patterns have the potential to act as biomarkers for the diagnosis of a target disease^28, 29^. In previous studies, WFA was the most feasible lectin probe for detecting liver cancer specific glycosylation changes in bile and serum^16^. Although expression of sialylated MUC1 was detected in cancer and non-cancer specimens, WFA-enriched sialylated MUC1 was evident only in the cancer specimens^16^. For these reasons, good diagnostic performance of WFA-sialylated MUC1 as a glycomarker of liver cancer was achieved by the detection of liver-specific glycan changes in serum sialylated MUC1 using WFA lectin and WFA-sialylated MUC1 was shown in this study to be a novel biomarker of a subtype of HCC with HPC/biliary features.

All our study patients underwent RFA for primary HCC. With the increase in the aging population worldwide, minimally invasive therapy is required more and more, particularly for elderly patients. While liver resection is recommended as the first-line therapy for patients with a small HCC^30, 31^, many patients cannot undergo surgery because of comorbidity or other complications. Therefore, RFA, a minimally invasive procedure, is increasingly important. Although RFA is usually carried out with curative intent, the recurrence of HCC after RFA is frequently observed. It is therefore important to identify those patients who have a high possibility of HCC recurrence after therapy with curative intent. Although whether RFA can be considered as a competitive alternative to resection is uncertain in early stage HCC, measurement of WFA-sialylated MUC1 may be helpful for selection of those patients suitable for RFA treatment. It has been reported that HCC patients transplanted beyond the Milan criteria without histochemical HPC feature achieved good survival, similar to those within the Milan criteria^13^. Therefore, WFA-sialylated MUC1 may support a limited expansion of liver transplantation indications. Furthermore, molecular classification of HCC could have clinical value as a predictive biomarker of drug response and selecting potential responders also in advanced stage of HCC^5^. In particular, HPCs in HCC tissue are considered a pivotal target for the eradication of cancer and detection of the subtype of HCC with HPC feature is important for the development of personalized and stratified clinical management^32^. In this manner, measurement of serum WFA-sialylated MUC1 may have a clinical impact, aiding the making of difficult therapeutic decisions.

The value of AFP and DCP as prognostic markers after curative therapy for HCC have been reported^33–35^. In this study, WFA-sialylated MUC1 levels increased independently of AFP and DCP levels and, hence, were an independent predictor of HCC recurrence by multivariate analysis. Therefore, WFA-sialylated MUC1 may be a useful complement to AFP and DCP as a prognostic marker for HCC recurrence.

The study has some limitations. AUROC of WFA-sialylated MUC1 for detection of sialylated MUC1 and CK19 staining was not high. It was because that the tumor specimens were obtained by needle biopsy and intratumoral heterogeneity may not be reflected in such specimens^36, 37^. In future studies, comparison between serum WFA-sialylated MUC1 levels and surgically obtained tumor samples is needed to evaluate the reproducibility cut off value of WFA-sialylated MUC1. Also, the number of tumor samples in the study was relatively low. These points may be clarified by a larger investigation. Anticancer therapy may rarely cause sarcomatous change of HCC and it had a poor prognosis^38^. Although RFA may had caused the change and affected recurrence, pathological examination at recurrence had not done, and a further investigation is needed.

In conclusion, serum WFA-sialylated MUC1 level was associated with HPC/biliary features in HCC and with a high incidence of tumor recurrence. It appears to be useful as a biomarker of HPC/biliary features in HCC and therefore for a predictor of recurrence after curative therapy.

## Methods

### Patients

One hundred and forty four consecutive patients with primary HCC, treated with RFA in Musashino Red Cross Hospital between January 2012 and January 2015, were enrolled in this study. All patients had presented BCLC stage 0 or A at entry, had imaging confirmation of complete ablation after RFA, and had been followed up for more than 6 months after curative therapy. Written informed consent was obtained from each patient. The study protocol was approved by the ethics review committees of Musashino Red Cross Hospital and conformed to the ethical guidelines of the Declaration of Helsinki.

### HCC diagnosis

HCC was diagnosed if tumors had early-phase vascular enhancement with late-phase washout on contrast-enhanced computed tomography (CT), magnetic resonance imaging (MRI), or angiography, according to the American Association for the Study of Liver Diseases, the European Association for the Study of the Liver and the Japan Society of Hepatology guidelines^30, 31, 39^. Of the 144 study subjects, 61 also had histopathologically confirmed HCC by ultrasound-guided biopsy, based on the World Health Organization criteria.

### Tumor biopsy and RFA methods, and follow up

All patients were treated by percutaneous RFA under ultrasound guidance. A needle-guiding technique was used, consisting of an initial guided needle and a secondary outer needle. This involves the initial insertion of a 21-gauge needle (Silux, Saitama, Japan) adjacent to the tumor under real-time US guidance, and using this to insert a 14-gauge Daimon outer needle (Silux), also adjacent to the tumor. After removal of the inner needle, an 18-gauge biopsy needle is inserted to obtain the tumor tissue sample. After removal of the biopsy needle, a 17-gauge cooled-tip electrode (Cool-Tip System, Valleylab, CO, USA) is inserted into the targeted tumor and ablation is performed. Dynamic CT or MRI was performed 1 to 2 days after RFA to evaluate the efficacy of ablation. Complete ablation of HCC was defined as non-enhancement of the lesion, including the entire surrounding liver parenchyma. RFA was repeated as needed until complete ablation was confirmed. To detect recurrence at an early stage, serum alpha-fetoprotein (AFP), lectin-reactive AFP (AFP-L3), and des-gamma carboxyprothrombin (DCP) levels were measured monthly, and dynamic CT or MRI was performed every 3 months after confirmation of cure. If HCC remained in contact with RFA scar at 3 month after primal RFA, it was judged as residual tumor and additional treatment was carried out. This was not counted as a recurrence. The evaluation for HCC recurrence was made using the same criteria as for primary lesions.

### Measurement of WFA-sialylated MUC1

An anti-sialylated MUC1 monoclonal antibody, MY.1E12, was used in this study^40^. For the measurement of serum WFA-sialylated MUC1 levels, a WFA-immobilized MY.1E12 sandwich ELISA was performed as described previously^18^. All specimens were diluted 1:10 with PBS containing 0.2% SDS and then heated at 95 °C for 5 min before the ELISA assay. The resulting solution (10 μL) was applied to the ELISA. All experiments were performed in triplicate and the mean value was used as the final value for each sample. The culture media of TGBC-1-TKB human gallbladder cancer cells were used as a standard for the measurement. Each value was calculated as a relative ratio to the standard curve. WFA-sialylated MUC1 values were expressed as μL of media/mL of serum (μL/mL).

### Immunohistochemical staining of liver biopsies

Formalin-fixed, paraffin-embedded sections of liver biopsies were autoclaved for 10 min at 110 °C in 10 mM citrate buffer (pH 6.0) for antigen retrieval, incubated for 30 min in 0.3% hydrogen peroxide/methanol for quenching endogenous peroxidase, blocked with 2.5% normal horse serum in PBS for 30 min at room temperature, and incubated with anti-sialylated MUC1 mouse monoclonal antibody (MY.1E12) diluted in 1% BSA/PBS (1:2000) for 60 min at room temperature. The primary antibody was detected using the ImmPRESS™ anti-mouse Ig reagent (Vector Laboratories, Burlingame, CA, USA) with diaminobenzidine. The sections were counter-stained with hematoxylin. Staining of liver biopsies with anti-CK19 mouse monoclonal antibody (RCK108; Dako, Glostrup, Denmark) was performed using a Bond-Max automated immunohistochemical staining machine (Leica Microsystems, Milton Keynes, UK) as previously reported^41^. Obvious staining in ≥1% of tumor cells was regarded as positive for sialylated MUC1 and CK19.

Fluorescent double-staining was performed using surgical resection specimens. The HCC tissue section was autoclaved for 10 min at 110 °C in 10 mM citrate buffer (pH 6.0), blocked with 2.5% normal horse serum for 30 min at room temperature, and probed with mouse anti-sialylated MUC1 monoclonal antibody (MY.1E12) and rabbit anti-CK19 monoclonal antibody (EP1580Y; Abcam) for 1 h at room temperature. The antibodies were detected with Alexa 488-conjugated anti-mouse IgG (ThemoFisher) and Alexa 594-conjugated anti-rabbit IgG (ThemoFisher) in 1% BSA/TBS supplemented with Hoechst 33342 (Molecular Probes), and visualized using an Axiovert 200 microscope and the ZEN software (Carl Zeiss).

### Statistical Analysis

Categorical data were compared using the chi-square and Fisher’s exact test. Distributions of continuous variables were analyzed using the Student’s *t* test or the Mann-Whitney *U* test. A p value of <0.05 was considered statistically significant. Receiver-operator characteristic (ROC) curves were constructed and optimal cut-off values were selected to maximize sensitivity, specificity, and diagnostic accuracy. The cumulative incidence of recurrence was determined by the Kaplan-Meier method, and differences among groups were assessed using a log-rank test. Factors associated with HCC recurrence were analyzed by the Cox proportional hazard model. Statistical analyses were performed using the Statistical Package for the Social Sciences software version 18.0 (SPSS Inc, Chicago, IL, USA).
